# Nutrition in Disguise: Effects of Food Neophobia, Healthy Eating Interests and Provision of Health Information on Liking and Perceptions of Nutrient-Dense Foods in Older Adults

**DOI:** 10.3390/foods10010060

**Published:** 2020-12-29

**Authors:** Olivia C. Romaniw, Ritika Rajpal, Alison M. Duncan, Heather H. Keller, Lisa M. Duizer

**Affiliations:** 1 Department of Food Science, University of Guelph, Guelph, ON N1G 2W1, Canada; oromaniw@uoguelph.ca; 2 Department of Human Health and Nutritional Sciences, University of Guelph, Guelph, ON N1G 2W1, Canada; rajpalr@uoguelph.ca (R.R.); amduncan@uoguelph.ca (A.M.D.); 3 Department of Kinesiology, University of Waterloo, Waterloo, ON N2L 3G5, Canada; hkeller@uwaterloo.ca; 4 Schlegel-University of Waterloo Research Institute for Aging, Waterloo, ON N2J 0E2, Canada

**Keywords:** older adults, nutrient-enhanced foods, food neophobia, healthy eating, health information, liking, sensory perceptions, check-all-that-apply (CATA)

## Abstract

Older adults (60+ years) are at higher risk of malnutrition. Improving the nutrient-density of their diets is important but presents challenges due to the introduction of new ingredients, liking implications and heterogeneity of older consumers. Ten nutrient-enhanced foods were evaluated for liking (9-point hedonic scale) and sensory perception (check-all-that-apply) by 71 older adults. Three foods were re-evaluated after participants were provided with information about their healthy ingredients and benefits. Participants were also segmented based on their degrees of food neophobia and interests in healthy eating, using questionnaires. The results showed that eight foods had adequate sensory appeal (overall hedonic score of ≥6) to be pursued for residential care menus. Segmentation based on food neophobia and healthy eating interests did not yield any meaningful differences between groups. The effect of health information on liking for the overall sample and subgroups was product-specific: liking scores only increased for the raspberry banana smoothie in the overall test population and higher healthy eating interest subgroup. Health information may lead to the experience of more positive attributes in some foods. Overall, eight foods that were tested could be accepted by a wide range of consumers and providing them with health information may further improve acceptance.

## 1. Introduction

Older adults (≥60 years old) are a significant and growing proportion of the Canadian population. In 2019, over 24% of Canadians fell into this age group, and current trends show that their proportion in the population will continue to grow rapidly in the upcoming years [1]. Older adults in Canada are also a vulnerable subgroup, with 34–68% at moderate to high risk of malnutrition [2]. This is due to factors such as physical difficulties, lack of interest in food, suppressed appetites and impaired tasting capabilities [3,4]. For these reasons, it is unlikely that older adults are meeting the necessary nutritional requirements, but offering nutrient-dense food options could help improve nutrient deficiencies and malnutrition in this population [5].

A prominent intervention method to improve the nutrient quality of older adults’ diets is through the development of nutrient-dense foods in a food-first approach to healthy aging [5]. However, improving the nutrient density of foods through nutritional enhancement comes with implications on liking, sensory perceptions and acceptance by consumers [6]. It may also involve the incorporation of ingredients that are nutritional, yet less familiar to older adults. Indeed, it has been shown that older adults have higher preferences for familiar foods [5,7] and are less likely to re-evaluate their life-long eating habits [8,9]. At the same time, research also shows that seniors are becoming increasingly interested in nutritional meal options that could help them maintain their health and independence as they age [10,11,12]. In fact, it has been reported that some older adults may be open to sacrificing taste for a healthier food product [13,14]. However, liking and sensory characteristics are still an extremely important factor in their food choice [15]. Therefore, creating a variety of nutrient-dense food choices that are well-liked by older consumers could help to address their malnutrition risk.

A challenge when developing food products for older adults is the high degree of heterogeneity between individuals [16]. Between the ages of 50 and 60 years, people have had a wide range of unique experiences that significantly shape their choices, values, motivations and lifestyles, and cause their nutritional needs to be similar but their food choices and preferences to be unrelated to one another [6]. Understanding motivations behind food choice, past experiences and future learning are important aspects to address in sensory research [17,18]. A potential strategy to investigate these differences is the separation of older consumers into smaller groups with more homogeneous attitudes and beliefs, or population segmentation. This could offer deeper insights into the potential success of new health products for this target market, determine products that would be acceptable to the population as a whole, and identify others that could be targeted to specific subsets of the population [6]. 

Over the past couple of decades, sensory science research, in conjunction with food product development practices, has been expanding beyond basic hedonic scores and sensory perception identification to consider factors that affect food perception and motivations behind food choice. Psychographic characteristics describe consumers’ psychological attitudes such as their personalities, values, opinions, interests and lifestyles habits, or, in this case, their general beliefs and motives regarding food [6]. Two important food-related psychographic factors in sensory research are food neophobia and general interests in healthy eating. 

Food neophobia is the fear of consuming novel foods and is typically characterized by the avoidance of new ingredients and products [19]. For older adults, food neophobia has been identified as a factor that could adversely impact willingness to try and the realistic consumption of new, healthy food products, including functional foods [20]. A questionnaire developed by Pliner and Hobden [19] can be used to explore relationships between food neophobia, liking of food and sensory perceptions. Furthermore, the general health interests of consumers can be explored using a subscale of the Health and Taste Attitude Survey developed by Roininen, Lähteenmaki and Tuorila [21]. Although this subset of the questionnaire has been used very often in literature to describe and characterize a certain study population, and is proven to be a strong indicator of eating patterns [22], it has not yet been used to investigate differences in sensory perceptions of foods, and its applications for food acceptance are also quite limited. Moreover, neither food neophobia nor general interests in healthy eating have been tested in strictly older populations.

Furthermore, since older adults have, in theory, demonstrated more willingness to try healthier foods and greater interest in functional meal options [23], receiving information on the healthy ingredients and nutritional benefits of a food product could have practical impacts on their liking and perceptions. The effect of this type of information is well-documented yet conflicting in literature. It has only been used once specifically with older adults to study the effects of information regarding salt reduction on liking of meatballs and protein-enhancement on bread [24]. Liking scores for these products increased after receiving information about their positive nutritional changes [24].

The primary objective of this study was to investigate the liking and sensory perceptions of ten nutrient-dense foods tailored for older adults to determine their feasibility for improving the nutrient quality of older adults’ diets. A second objective was to observe the impacts of food neophobia and healthy eating interest segmentation on liking scores and perceptions of the foods. The final objective was to examine the influence of health information on sensory evaluations for both the overall participant sample and the food neophobia and healthy eating interest segmented groups. It was hypothesized that lower food neophobia and higher interests in healthy eating would lead to higher liking scores as well as the expression of more positive sensory attributes. It was also hypothesized that the provision of information on the healthy ingredients and the benefits for older adults would increase liking scores, but affect subgroups differently; specifically, more significant increases in liking were expected to be seen in the lower food neophobic and high health interest subgroups.

## 2. Materials and Methods 

### 2.1. Food Products

Twenty-one nutrient-enhanced food products were developed by chefs in residential care homes from Guelph and Waterloo, Ontario, Canada. Key healthy ingredients were identified based on their content of vitamins and minerals noted to be often lacking in the diet of older adults [25] and their feasibility of incorporation into foods preferred by older adults (e.g., desserts, baked products and soups). A bench-top tasting session was conducted with 19 people involved in the project to assess suitability of the 21 food products for incorporation into a residential care menu. From the 21 foods, 10 were selected for sensory evaluation based on their acceptability and nutrition profile. These products included: apple cider muffin, beef and barley soup, cheese and spinach quiche, cranberry almond streusel with yogurt, lentil brownie, mulligatawny soup, oatmeal berry parfait, orange carrot muffin, raspberry banana smoothie, and tomato cream cheese and wild rice soup. All of these products were made with healthy and novel ingredients (Table 1) and their nutrient composition can be found in a previous publication [25]. For sensory testing, all foods were prepared by the researchers in a food-grade laboratory at the University of Guelph (Guelph, ON, Canada). 

### 2.2. Panelists

This research was approved by the Research Ethics Board of the University of Guelph (REB#18-03-012). Seventy-one older adults (73.2% female) participated in sensory testing. They were recruited from Guelph, Canada using posters, recruitment kiosks and email correspondence with recreational organizations and clubs for older adults. All panelists participating were at least 60 years of age (average ± SD age = 71.1 ± 6.4 years) and community-dwelling with no sensitivities, food allergies, special diets or swallowing difficulties that would have prevented them from consuming any of the foods. They were also screened to ensure that they cooked a main meal in their household at least twice per week to confirm they were regularly exposed to a variety of different food products. 

### 2.3. Sensory Testing

Participants visited the University of Guelph Food Science Building for sensory testing once per week for four consecutive weeks. During each visit, they were given three or four foods to taste and evaluate (Table 2). Foods were served to panelists at appropriate temperatures, with baked goods and streusel at room temperature, quiche (40 °C) and soups (60 °C) at a warm temperature, and smoothie (−14 °C) and parfait (4 °C) at a cold temperature (Table 2). All foods were served in 118-millilitre Styrofoam cups with lids, and labelled with either unique, three-digit codes (Weeks 1–3) or the generic name of the product (Week 4). All testing sessions were completed one week apart. The order in which the samples were presented and tasted each week was randomized among participants to reduce bias, but all participants tasted the same samples each week. Panelists cleansed their palates with table crackers and water between tasting each sample.

For each sample, participants were asked to consume enough of the food to be able to make an accurate assessment, and then respond to a series of questions relating to their liking of the appearance, flavor and texture of the food, as well as their overall liking, on a 9-point hedonic scale ranging from “1 = dislike extremely” to “5 = neither like nor dislike” to “9 = like extremely”. They were also asked whether they were likely to consume the product again and given space for optional open comments. Finally, a check-all-that-apply (CATA) exercise [26] was completed with a pre-determined list of flavors (bitter, bland, earthy, fresh, salty, sour, spicy, stale and sweet) and texture terms (chunky, creamy, dense, doughy, dry, grainy, moist, powdery, slippery, smooth, soft, sticky, thick and thin) developed by the researchers. Panelists selected all the attributes they perceived while processing each sample. CATA terms for both categories were listed in a randomized order for each panelist and for each session.

During the first three weeks of testing, the participants were blind to any information about the foods other than the product category into which they fell. For the fourth and final visit, three foods (lentil brownies, mulligatawny soup and raspberry banana smoothie) were selected to be re-tasted and re-evaluated to assess the effects of health information on liking and sensory perceptions of these nutrient-enhanced foods. These foods were chosen because they represented all product categories and their nutrient-dense ingredients were sufficiently hidden in the appearance of the food so it was not easy for panelists to determine their ingredients by observation and/or tasting. Before tasting each sample, participants were given an information sheet that outlined the name of the product, each healthy/novel ingredient in its recipe, and the potential health benefits of each ingredient relevant to older adults (Table S1). Then, after re-tasting the sample, panelists completed the same sensory questionnaire used in previous sessions.

At the end of the third meeting after blind evaluations, the participants completed the published Food Neophobia Scale questionnaire developed by Pliner and Hobden [19], which involves rating agreeability with ten statements on a 7-point scale, ranging from “1 = disagree strongly” to “4 = neither agree nor disagree” to “7 = agree strongly” to determine their degree of food neophobia. To determine participants’ interests in healthy eating, they completed the General Health Interest sub-scale questionnaire from the larger survey developed and published by Roininen et al. [21] with one additional question regarding the likelihood to accept healthy foods if they tasted worse than regular foods. The same agreeability scale as the Food Neophobia Scale was used for the nine unique statements and the questionnaire with the additional question tested statistically with SAS^®^ statistical software and Cronbach’s alpha test to ensure internal reliability of the scale.

### 2.4. Data Analysis

#### 2.4.1. Sensory Evaluation—Overall Participant Sample

Prior to data analysis, the distribution of hedonic score counts for each food were examined to determine the presence of any split populations in liking that could affect the results. Following this, analysis of variance (ANOVA) and Tukey’s Honestly Significant Difference (HSD) mixed model tests (α = 0.05) were performed using SAS^®^ software (Version 9.4, 2019) to analyze hedonic scores for appearance, flavor, texture and overall liking from the blind sensory testing for each of the ten nutrient enhanced food products. To compare overall liking scores before and after provision of information about the nutrient-dense ingredients and potential benefits, *t*-tests (α = 0.05) were also performed using SAS^®^ software to compare overall liking scores before and after participants received this information for the three foods. 

To analyze the likelihood of consuming each product again, a chi-square frequency test was performed using SAS^®^ software to determine the number of times each response was reported per food product. Percentages and differences in the proportion of responses before and after receiving ingredient information were calculated using SAS^®^ software.

The open-ended comments were consolidated and summarized to investigate any recurring themes.

To develop a sensory profile of each food, the results of the CATA exercise from the blind evaluation were analyzed on XLSTAT software. Cochran’s Q test for multiple pairwise comparisons (α = 0.05) was completed to determine the proportion of responses per attribute per food product. To visualize the data yielded from this test, a symmetric plot was generated to demonstrate how the ten products were aligned with each other and with the sensory terms based on the attributes most expressed by the participants. Furthermore, a penalty analysis test was used to show which attributes that had a significant impact on overall liking. Cochran’s Q test was also used to compare attribute expression proportions between the blind and informed evaluations of the three foods (α = 0.05).

#### 2.4.2. Segmented Sample

To segment the participants based on an individual’s degree of food neophobia, the sum score of the ten questions from the Food Neophobia Scale was calculated and participants were divided at the median score into either a high food neophobia (HFN) group (*n* = 35, score range = 31–48) and low food neophobia (LFN) group (*n* = 36, sum score = 16–30). The same procedure was followed for the General Health Interest sub-scale to divide the population into those with higher interests in healthy eating (HIH; *n* = 36, sum score range = 46–62) and lower interests (LIH; *n* = 35, score range = 22–45). This method of population segmentation for these questionnaires was modelled after Laaksonen, Knaapila, Niva, Deegan and Sandell [27], who separated their participant sample into three approximately evenly sized groups based on sum scores. However, due to a lower number of participants in this study, our sample was only segmented at the median to achieve two groups.

To determine any significant differences in liking between sample population segments, *t*-tests (α = 0.05) were performed using SAS^®^ software to compare overall liking scores between the HFN and LFN groups, and between the HIH and LIH groups for each food. Furthermore, *t*-tests (α = 0.05) were also done to compare overall liking scores before and after receiving health information for each subgroup to investigate any disparities from trends seen in the overall population. 

Cochran’s Q test on XLSTAT software was performed for CATA responses for all ten foods to compare selection proportions between HFN and LFN groups and HIH and LIH groups (α = 0.05).

## 3. Results

### 3.1. Sensory Evaluation for Overall Participant Sample

No split populations were observed in liking responses. The mean liking scores for appearance, flavor, texture and overall liking for each of the ten foods assessed during blind sensory evaluation are summarized in Table 3. The cheese and spinach quiche, oatmeal berry parfait and lentil brownies received the highest liking scores in most categories. These three foods also had the highest percentages of participants indicating that they were likely to consume the food again (cheese and spinach quiche-91%, oatmeal berry parfait-73% and lentil brownies-86%). The raspberry banana smoothie and tomato cream cheese and wild rice soup were the least liked and, consequently, had the highest proportion of participants respond that they would not consume these foods again, being 40% and 37% respectively. Common complaints regarding the raspberry banana smoothie were that it contained seeds (raspberries) that got stuck in their teeth and dentures, and that it tasted medicinal and artificial. 

**Figure 1:**
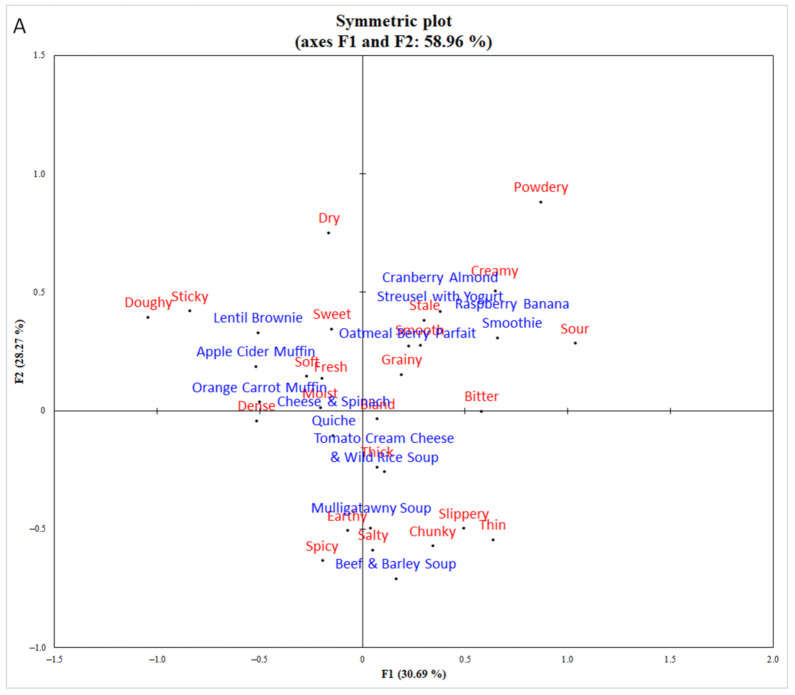

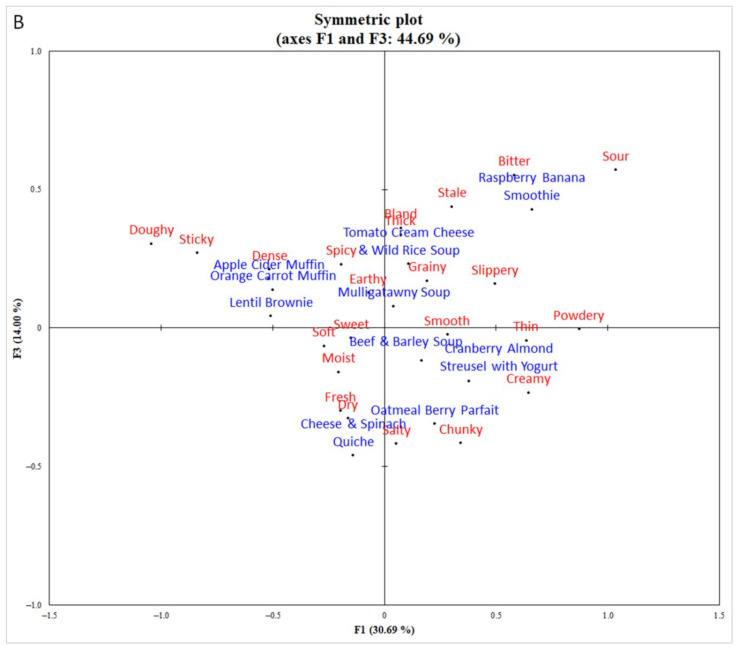
Symmetric plot derived from check-all-that-apply (CATA) responses of ten nutrient-dense foods during sensory evaluation without nutrition and health information. (**A**) Factors 1 and 2 explained 59.0% of the variability. (**B**) Factors 1 and 3 explained 45.0% of the variability.

**Figure 2:**
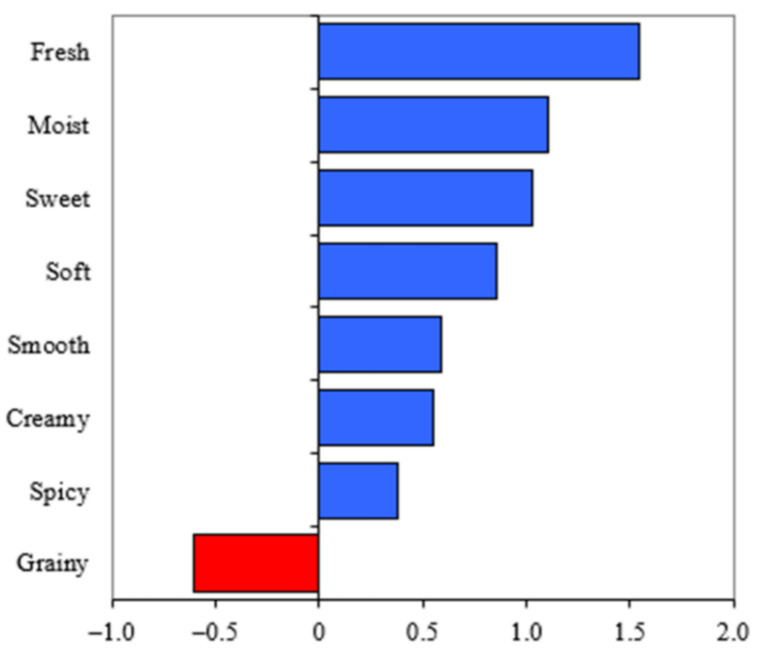
Attribute mean impact on overall liking for ten nutrient-dense foods derived from penalty analysis (α = 0.05).

The symmetric plot in Figure 1 shows each food and its relationships to the sensory attributes from the CATA exercise, as well as the products’ relationships to each other. The first two factors explained 59% of data variability, which is comparable to Ares et al. [26], where specific groupings were observed based on product-type. The ten foods were grouped as baked products (two muffins and lentil brownie), three soups, and foods containing prominent dairy products (raspberry banana smoothie, oatmeal berry parfait and cranberry almond streusel with yogurt), with the cheese and spinach quiche in its own quadrant. The penalty analysis test showed that overall liking was positively affected by fresh, moist, sweet, soft, smooth, creamy and spicy perceptions, and negatively impacted by graininess (Figure 2). 

Provision of information on nutrient-dense ingredients incorporated into the recipes and their potential health benefits for the consumer significantly increased overall liking scores for the smoothie and brownie, but not for mulligatawny soup (Table 4). Furthermore, the health information increased the likelihood that the participants would consume the product again for all three foods, but most prominently for the raspberry banana smoothie. Health information also changed the expression of certain sensory perceptions. After reading the information about the raspberry banana smoothie recipe, sweet, sour and smooth sensations increased, and blandness decreased. For the mulligatawny soup, thickness and freshness increased, and salty and slippery proportions were less expressed. No changes in sensory perceptions were observed in the lentil brownies. 

### 3.2. Segmented Samples

**Figure 3:**
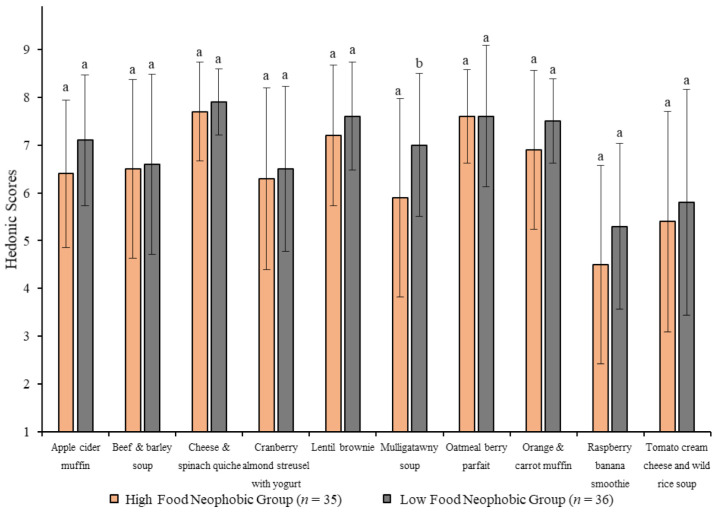
Means and standard deviations for overall liking of ten nutrient-dense foods between food neophobia subgroups without health and nutrition information. Y-axis represents the 9-point hedonic scale (1 = dislike extremely, 5 = neither like nor dislike, 9 = like extremely). For each food, values with different letters are significantly different (*p* < 0.05). *n* = 34 for cheese and spinach quiche and tomato cream cheese and wild rice soup in high food neophobia group due to missing data.

Participants with a lower degree of food neophobia (i.e., those who are more willing to try novel foods, LFN) showed significantly higher overall liking scores for the mulligatawny soup only (Figure 3). There were no other significant differences in overall liking during the blind sensory evaluations. In terms of sensory perceptions, the high food neophobia (HFN) group found the lentil brownies to be more dry. Finally, provision of health information increased the overall liking scores for both groups for the raspberry banana smoothie and lentil brownies, but not for the mulligatawny soup (Table 5), which follows the same pattern seen in the overall sample.

**Figure 4:**
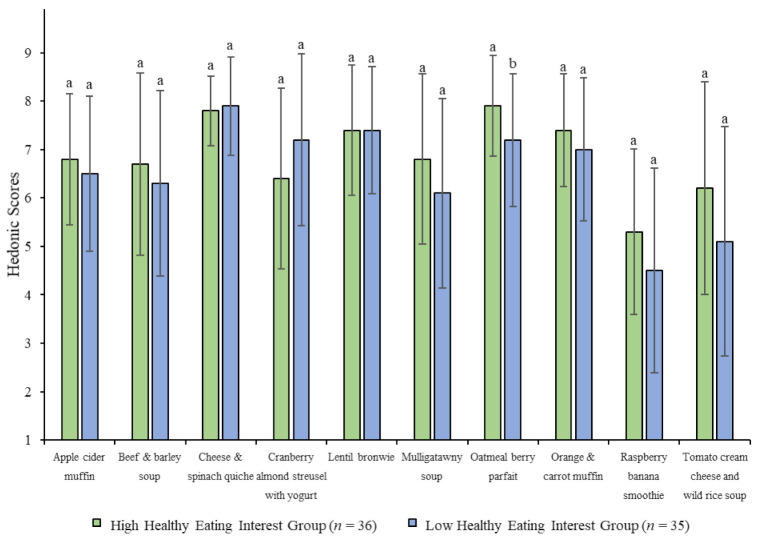
Means and standard deviations for overall liking of ten nutrient-dense foods between healthy eating interest subgroups without health and nutrition information. Y-axis represents the 9-point hedonic scale (1 = dislike extremely, 5 = neither like nor dislike, 9 = like extremely). For each food, values with different letters are significantly different (*p* < 0.05). n = 34 for cheese and spinach quiche and tomato cream cheese and wild rice soup in low healthy eating interest group due to missing data.

Participants who were more health conscious (HIH group) reported higher overall liking scores for the oatmeal berry parfait than the less health conscious consumers (LIH group), but no other significant differences were observed (Figure 4). Additionally, this study did not reveal any significant differences in sensory perceptions of the foods between the two health interest groups. However, the LIH group differed from the HIH group and overall population in that their overall liking scores of the raspberry banana smoothie significantly increased after being given the nutrition and health information. On the other hand, the HIH group demonstrated significantly higher liking scores for the mulligatawny soup after reading the health information, but the LIH group and overall sample did not. Finally, while nutrition and health information significantly increased liking of the lentil brownie for the LIH group and overall sample, it did not have a significant effect on the HIH group’s overall liking scores (Table 6).

## 4. Discussion

Based on the overall liking score (≥6, Table 3), this study identified up to eight newly developed, nutrient-enhanced food products that could be used to increase the nutrient density of older adults’ diets and be a part of the food-first intervention to combatting malnutrition in this population. Using the 9-point hedonic scale, two products, the cheese and spinach quiche and the oatmeal berry parfait, were liked very much overall, and received high scores for flavor and texture. The quiche was also associated with freshness, and the parfait with smoothness and freshness, which are positive attributes. However, their appearance scores were slightly lower than the ratings of the other categories. For the quiche, this drop could possibly be explained by the appearance of the spinach and kale ingredients seen on top of the product. For the parfait, some participants reported that the pudding layer looked slightly green, which can be addressed for improvement in later formulations. Additionally, the parfait was perceived as grainy, an attribute shown to have a negative impact on liking, but this is likely due to the baked oats and seeds that make up the base layer of the parfait. Given the high overall liking scores, these foods can be targeted for future implementation in residential home menus.

Furthermore, five of the foods were rated as moderately liked, including the lentil brownie, orange and carrot muffin, apple cider muffin, beef and barley soup and mulligatawny soup. Generally, these foods received consistent liking scores for appearance, flavor, texture and overall liking, and are all associated with some of the attributes shown to drive liking: the baked goods were perceived as fresh, moist, sweet and soft, while the soups were closely related to spicy. It is important to note a potential explanation for both soups’ lower liking ratings for appearance is the kale powder used in the formulation, which gives the samples a distinct green color. All of these nutrient-dense products will also be proposed for residential care menus.

The cranberry almond streusel with yogurt and tomato cream cheese and wild rice soup were liked only slightly. Although the streusel received low liking scores for its appearance, texture and overall liking, and was perceived as grainy, the flavor was still liked moderately, and even received the fifth highest flavor score of the ten foods. Therefore, it is suggested that this streusel could be incorporated into different foods such as healthy baked goods instead of yogurt to potentially mask its more undesirable appearance and texture while enhancing a food product’s flavor and providing older adults with a nutrient-dense food option. Further formulation testing and sensory evaluation is recommended. In terms of the tomato cream cheese and wild rice soup, it is being eliminated from future testing and menu implementation due to its low acceptance scores in all sensory categories.

Finally, the raspberry banana smoothie was neither liked nor disliked by this population, who cited that raspberry seeds were getting stuck in their dentures and that the flavor tasted artificial and medicinal. It was also perceived to have an undesirable grainy quality. It received low acceptance ratings in all four sensory aspects that were tested and will therefore also be removed from any future applications of these foods.

Segmenting the participants into groups with varying degrees of food neophobia revealed a significant difference in liking for the mulligatawny soup only; the group with lower degrees of food neophobia liked the soup more. Interestingly, this product was the only food that was internationally inspired. The other nine foods used in this study incorporated flavors typically found in Western foods, while the mulligatawny soup contained spices that may be more unfamiliar to a Canadian population. This finding is consistent with literature that states that food neophobia only influences liking of unfamiliar food products [28,29,30] but contradicts Januszewska and Viaene [31] who concluded that food neophobia affects liking of familiar, culturally local foods as well. Furthermore, while some changes in sensory perceptions were observed between groups for certain foods, these findings were too limited to suggest any concrete relationships between food neophobia and sensory perceptions of these nutrient-enhanced products.

The group of participants that was shown to have a greater interest in healthy eating reported significantly higher liking scores for only the oatmeal berry parfait than the group with lower interests in healthy eating. Although this is likely not a very significant finding, it is hypothesized that the higher health interest group may be more accustomed to tastes and textures of the nutritious ingredients incorporated into this traditional dessert, such as plain Greek yogurt, berries, oats and seeds, since it is assumed that a greater interest in healthy diets is correlated with the consumption of a healthier diet itself [22]. Overall, these findings suggest that the influence of older adults’ healthy eating interests on liking of nutrient-enhanced foods is product-specific. This conclusion is consistent with research conducted by Laaksonen et al. [27], although they found more significant effects in less-liked foods, which is different than this study, since the parfait was a well-liked product in general. It is also important to note that there were no significant differences in sensory perceptions between groups for any of the ten foods so, while this psychographic factor may affect liking of certain products, it may not impact the sensory attributes perceived by older adults.

The effects of provision of information of the nutritious ingredients in the food products and their potential health benefits on liking was product-specific. For the total test population, hedonic scores for the lentil brownie and raspberry banana smoothie significantly increased, while those for the mulligatawny soup did not. However, receiving the nutrition and health information did increase the likelihood that all three foods would be consumed again, with the largest change in proportion observed in the raspberry banana smoothie (25.6% increase). Furthermore, provision of information increased the expression of sensory perceptions with a positive relationship to liking, such as freshness in the mulligatawny soup, and sweetness, sourness and smoothness in the raspberry banana smoothie. The smoothie was also perceived as less bland after information was provided. Recent literature has shown that product information could shift attribute perceptions towards more positive and less negative characteristics, but these findings are also product-specific [32]. Interestingly, the mulligatawny soup was perceived as less salty after participants were told that it was made with a low-sodium broth. Reis, Alcaire, Deliza and Ares [33] found a similar effect on sweetness when assessing the effect of product information on sugar-reduced or alternatively sweetened fruit juices. In all, providing the general population of older adults with information regarding the nutrition of healthier food products could increase liking, drive them to eat the nutritious food options more frequently, and perhaps lead them to experience more positive and less negative sensory perceptions while consuming these foods.

Although the liking scores of the food neophobia subgroups were affected by health information in the same way as the overall population, the varying degrees of interest in healthy eating showed some trends. The raspberry banana smoothie was clearly more liked by the high healthy eating interest group after receiving information about the healthiness of the ingredients. Villegas, Carbonell and Costell [34] observed the same effect with milk and soy beverages. Although this smoothie was eliminated from future testing after the blind sensory evaluation, the informed testing revealed that there could be a market for this food product with older adults who are interested in healthy food options when appropriate health information is clearly presented to them. Further testing with this demographic in these conditions would be necessary. The different trends observed in the healthy eating interest subgroups’ liking scores before and after receiving health information for the lentil brownie and mulligatawny soup were likely just an artefact of their standard deviations since the actual differences between average hedonic ratings were minimal. Overall, these findings contribute to the conclusion that older adults with information about the healthiness of a food may affect liking, but this depends on the food product and, in some cases, the certain attitudes of the population itself.

A strength of this study is that the sensory testing was performed with the specific target consumers of older adults. Additionally, in the participant screening process, some potential variability was controlled by ensuring that older adults were community-dwelling and regularly ate home-cooked meals. This way, it is more likely they had more recent exposure and experience eating a variety of foods and ingredients than older adults in residential care, who often order from a limited menu. Steps were also taken when conducting sensory testing to reduce bias, such as sample randomization and blinding. The nutrient-dense foods tested in this study were designed by chefs in residential care homes who regularly create meals for and interact with older adults, and they chose to nutritionally enhance food products historically enjoyed by this consumer group. Therefore, these foods meet the needs and preferences of a typical older adult consumer and have significant potential to be incorporated into their diet. Finally, the Food Neophobia and General Health Interest questionnaires offer the advantage of being well-validated and successfully used many times in literature, therefore supporting their use in this research as well.

A limitation of this study is the sample size; participant segmentation is often performed with a greater number of panelists. The sample size for this study was limited by time and resources; given the multiple products and test outcomes evaluated. Furthermore, due to the considerable diversity of food types and food product categories in this study, it is difficult to gain a comprehensive understanding of the attribute perceptions of each food using a CATA technique. Since a limited number of terms can only be included in the pre-determined list of attributes without causing confusion and fatigue during sensory evaluation, it was not possible to provide a more representative profile of the foods using a rapid profiling method. Descriptive sensory analysis in the form of a trained panel would have provided more insight into the sensory properties of the foods, but this method was not worthwhile given the large number of samples and relative value of the information.

## 5. Conclusions

This study identified up to eight newly developed nutrient-dense food products that could be used in a food-first approach to improve the nutrient quality of older adults’ diets based on their sensory appeal, healthy ingredients and functionality for this demographic. These foods have the potential to be implemented in residential care menus and could be accepted by older consumers with varying degrees of food neophobia and interests in healthy eating since neither of these psychographic characteristics had a large effect on overall liking of the foods or sensory perception. Providing participants with information about the nutrient-dense ingredients incorporated into the foods and their potential health benefits could improve the acceptability of the foods and promote the perception of more positive sensory attributes and decrease the expression of certain negative attributes. However, these effects are product-specific. Finally, although it was initially determined that the raspberry banana smoothie was not liked enough by the overall panelists to be pursued for further testing in residential care home menus, the informed evaluation revealed that it could be accepted by older adults who are interested in healthy eating when accompanied with health information. This finding suggests that there may in fact be a market for this less liked food product. More in-depth research into the segmentation of older consumers and the effects of provision of health information on liking and sensory perceptions is needed to understand if there is a relationship between these two concepts, and could lead to a better understanding of how to create foods for older adults.

## Figures and Tables

**Table 1:** Nutrient-dense ingredients in ten foods developed and used for sensory analysis.

Food	Nutrient-Dense Ingredients
Apple cider muffin	Bran, cinnamon, Greek yogurt, wheat germ
Beef and barley soup	Chia seeds, flax seeds, hemp seeds, kale powder, turmeric
Cheese and spinach quiche	Cheddar cheese, kale, skim milk powder, spinach
Cranberry almond streusel with yogurt	Chia seeds, cinnamon, dried cranberries, ground almonds and almond slivers, hemp seeds, wheat germ
Lentil brownie	Lentils
Mulligatawny soup	Chia seeds, cinnamon, flax seeds, ginger, hemp seeds, kale powder, lentils, turmeric
Oatmeal berry parfait	Cinnamon, Greek yogurt, hemp seeds, oats, raspberries, skim milk powder
Orange carrot muffin	Bran, cinnamon, turmeric, wheat germ
Raspberry banana smoothie	Cinnamon, Greek yogurt, hemp oil, raspberries, skim milk powder, turmeric
Tomato cream cheese and wild rice soup	Kale, kale powder, lentils, turmeric

**Table 2:** Serving size and temperature of ten nutrient-enhanced foods used in sensory evaluation.

Week	Food Category	Nutrient-Enhanced Foods	Serving Size (g)	Serving Temperature (°C)
1	Breakfast and lunch	Apple cider muffin	19 ± 1.6	Room
		Cheese and spinach quiche	32 ± 3.4	40 ± 2
		Orange carrot muffin	18 ± 1.4	Room
		Raspberry banana smoothie	51 ± 7.7	4 ± 2
2	Soups	Beef and barley soup	45 ± 1.1	60 ± 2
		Mulligatawny soup	46 ± 1.7	60 ± 2
		Tomato cream cheese and wild rice soup	45 ± 2.2	60 ± 2
3	Desserts	Cranberry almond streusel with yogurt	9 ± 0.5	Streusel: Room Yogurt: 4 ± 2
		Lentil brownie	25 ± 2.1	Room
		Oatmeal berry parfait	28 ± 1.3	4 ± 2
4	Re-evaluation after provision of health information	Lentil brownie	Included above	Room
		Mulligatawny soup	Included above	60 ± 2
		Raspberry banana smoothie	Included above	4 ± 2

**Table 3:** Mean hedonic scores for sensory evaluation without nutrition and health information about nutrient-dense foods. A 9-point hedonic scale was used (1 = dislike extremely, 5 = neither like nor dislike, 9 = like extremely).

Food	Appearance	Flavor	Texture	Overall
Apple cider muffin	7.2 ± 1.33 ^a^*^†^	6.8 ± 1.37 ^a†^	7.1 ± 1.30 ^a†^	6.7 ± 1.49 ^a^
Beef and barley soup	6.2 ± 1.81 ^bc^	6.6 ± 1.86 ^a^	6.5 ± 1.81 ^a^	6.5 ± 1.88 ^a^
Cheese and spinach quiche	7.5 ± 1.39 ^d†^	7.9 ± 0.87 ^bc†^	7.8 ± 0.96 ^cde†^	7.8 ± 0.87 ^de†^
Cranberry almond streusel with yogurt	5.9 ± 1.65 ^a^	6.8 ± 1.98 ^abc^	6.0 ± 2.07 ^ab^	6.3 ± 1.80 ^abc^
Lentil brownie	7.7 ± 1.09 ^ab^	7.5 ± 1.28 ^bc†^	7.5 ± 1.28 ^ab^	7.4 ± 1.32 ^bcd^
Mulligatawny soup	5.9 ± 1.89 ^cd^	6.5 ± 1.98 ^c^	6.5 ± 1.84 ^bc^	6.5 ± 1.89 ^cd^
Oatmeal berry parfait	6.7 ± 1.96 ^cd^	7.7 ± 1.28 ^cd^	7.5 ± 1.38 ^bcd^	7.6 ± 1.26 ^cd^
Orange and carrot muffin	7.5 ± 1.13 ^a^	7.2 ± 1.26 ^ab^	7.2 ± 1.17 ^a^	7.2 ± 1.34 ^ab^
Raspberry banana smoothie	5.6 ± 1.91 ^e^	4.9 ± 2.10 ^d^	5.5 ± 2.06 ^de^	4.9 ± 1.94 ^ef^
Tomato cream cheese and wild rice soup	4.8 ± 2.25 ^d^	5.7 ± 2.30 ^e†^	5.8 ± 2.07 ^e^	5.6 ± 1.80 ^f†^

* Values within a column with different letters are significantly different (*p* < 0.05; Tukey’s HSD). ^†^ *n* = 70 due to missing data (all other values *n* = 71).

**Table 4:** Mean hedonic scores for overall liking from sensory evaluation with and without information on the nutrient-dense ingredients and potential health benefits of three foods. A 9-point hedonic scale was used (1 = dislike extremely, 5 = neither like nor dislike, 9 = like extremely).

Food	Overall Liking Score without Information	Overall Liking Score with Information	Difference between Means	Increase (%) in Consumption Likelihood after Informed Health Benefits
Lentil brownie	7.4 ± 1.32 ^a^*^,†^	7.9 ± 0.94 ^b^	0.5	8.6
Mulligatawny soup	6.5 ± 1.89 ^a^	6.8 ± 1.80 ^a†^	0.3	6.2
Raspberry banana smoothie	4.9 ± 1.94 ^a^	6.0 ± 2.12 ^b^	1.1	25.6

* Values within a row with different letters are significantly different (*p* < 0.05). ^†^ *n* = 69 due to missing data (all other values *n* = 71).

**Table 5:** Food neophobia subgroup differences on mean overall liking with and without information on nutrient-dense ingredients and health benefits for three foods. A 9-point hedonic scale was used (1 = dislike extremely, 5 = neither like nor dislike, 9 = like extremely).

Food	Group	Overall Liking Score without Information	Overall Liking Score with Information	Difference between Means
Lentil brownie	HFN (*n* = 35)	7.2 ± 1.47 ^a^*	7.8 ± 1.05 ^b^	0.6
	LFN (*n* = 36)	7.6 ± 1.13 ^a^	7.9 ± 0.85 ^b^	0.3
	Total sample (*n* = 71)	7.4 ± 1.32 ^a^	7.9 ± 0.94 ^b^	0.5
Mulligatawny soup	HFN (*n* = 35)	5.9 ± 2.08 ^a^	6.2 ± 2.03 ^a^	0.3
	LFN (*n* = 36)	7.0 ± 1.50 ^a^	7.3 ± 1.41 ^a^	0.3
	Total sample (*n* = 71)	6.5 ± 1.89 ^a^	6.8 ± 1.80 ^a^	0.3
Raspberry banana smoothie	HFN (*n* = 35)	4.5 ± 2.08 ^a^	5.5 ± 2.19 ^b^	1.0
	LFN (*n* = 36)	5.3 ± 1.74 ^a^	6.4 ± 1.93 ^b^	1.1
	Total sample (*n* = 71)	4.9 ± 1.94 ^a^	6.0 ± 2.12 ^b^	1.1

HFN = high food neophobia group, LFN = low food neophobia group. * Values within a row with different letters are significantly different (*p* < 0.05).

**Table 6:** Healthy eating interest subgroup differences on mean overall liking with and without information on nutrient-dense ingredients and health benefits for three foods. A 9-point hedonic scale was used (1 = dislike extremely, 5 = neither like nor dislike, 9 = like extremely).

Food	Group	Overall Liking Score without Information	Overall Liking Score with Information	Difference between Means
Lentil brownie	HIH (*n* = 36)	7.4 ± 1.34 ^a^*	7.9 ± 1.09 ^a^	0.5
	LIH (*n* = 35)	7.4 ± 1.31 ^a^	7.8 ± 0.76 ^b^	0.4
	Total sample (*n* = 71)	7.4 ± 1.32 ^a^	7.9 ± 0.94 ^b^	0.5
Mulligatawny soup	HIH (*n* = 36)	6.8 ± 1.76 ^a^	7.2 ± 1.30 ^b^	0.4
	LIH (*n* = 35)	6.0 ± 1.99 ^a^	6.2 ± 2.07 ^a^	0.2
	Total sample (*n* = 71)	6.5 ± 1.89 ^a^	6.8 ± 1.80 ^a^	0.3
Raspberry banana smoothie	HIH (*n* = 36)	5.3 ± 1.71 ^a^	6.8 ± 1.72 ^b^	1.5
	LIH (*n* = 35)	4.5 ± 2.12 ^a^	5.1 ± 2.17 ^a^	0.6
	Total sample (*n* = 71)	4.9 ± 1.94 ^a^	6.0 ± 2.12 ^b^	1.1

HIH = high interest in healthy eating group, LIH = low interest in healthy eating group. * Values within a row with different letters are significantly different (*p* < 0.05).

## Data Availability

The data presented in this study are available on request from the corresponding author. The data are not publicly available due to privacy concerns.

## Supplementary Materials

The following are available online at https://www.mdpi.com/2304-8158/10/1/60/s1, Table S1: Summary of information provided to participants on healthy/novel ingredients in three foods and their potential health benefits during informed sensory evaluation.
