# Supplementary material for: Nutrition in Disguise: Effects of Food Neophobia, Healthy Eating Interests and Provision of Health Information on Liking and Perceptions of Nutrient-Dense Foods in Older Adults
Source: Foods. 2020 Dec 29;10(1):60. doi: 10.3390/foods10010060 (PMC7823595; doi:10.3390/foods10010060)
Supplement: Supplementary file 1 [file foods-10-00060-s001.pdf]

Table S1: Summary of information provided to participants on healthy/novel ingredients in three foods and their potential health benefits during informed sensory evaluation.

Food	Ingredient	Summary of Health Benefits
Lentil brownie	Lentil puree	• Contains protein, carbohydrates, fibre, vitamins, minerals and essential amino acids<br>• Lowers cholesterol, risk of heart disease and incidence of certain cancers<br>• Improves type 2 diabetes symptoms
	Olive oil as substitute for butter	• Healthier oil<br>• Lowers cholesterol, blood sugar, inflammation and swelling<br>• Antioxidants that protect organs from toxins<br>• May improve cognition and reduce risk of dementia<br>• Butter negatively impacts heart health and weight
Mulligatawny soup	Kale powder	• More concentrated and bioavailable nutrients in powder<br>• Contains vitamins A, C, K and folate, potassium, calcium, magnesium, protein (for muscle mass) and fibre (for gut, heart and immune health)<br>• Antioxidants to protect organs from toxins<br>• May reduce risk of chronic disease
	Lentils	• Contains protein, carbohydrates, fibre, vitamins, minerals and essential amino acids<br>• Lowers cholesterol, risk of heart disease and incidence of certain cancers<br>• Improves type 2 diabetes symptoms
	Flax seeds	• Contains omega-3's and -6's for brain and heart health<br>• Insoluble fibre for overall health<br>• May help metabolize certain hormones and have antioxidants
	Chia seeds	• Lower weight, triglyceride levels and blood sugar<br>• Contain omega-3's (for brain and heart health), protein/amino acids (muscle health) and fibre (overall health), and important minerals like calcium, phosphors and zinc (bone health)
	Hemp seeds	• Contain protein (for muscle health), fibre (overall health), B-vitamins (energy, metabolism and brain function), minerals like magnesium, phosphorus and potassium (bone health) and omega-3's and -6's (brain and heart health)
	Turmeric	• Contains curcumin antioxidant to reduce inflammation<br>• May increase short-term memory and prevent formation of brain tangles that cause dementia

	Cinnamon	• Contains polyphenols and cinnamaldehyde which prevent dementia<br>• Polyphenols reduce inflammation and protect against toxins<br>• Cinnamaldehyde prevents formation of brain tangles that cause dementia
	Low sodium broth	• Lowering sodium consumption helps with hypertension and reduces risk of stroke, heart disease, stomach cancer and osteoporosis<br>• Older adults are at-risk group for problems relating to salt intake
Raspberry banana smoothie	Raspberries	• Antioxidants that protect against toxins and reduce inflammation that could damage muscles and organs
	Greek yogurt	• Contains probiotics (for digestive and immune health) and different nutrients (physical and mental health)<br>• Lower risk of osteoporosis, muscle mass loss, frailty and depression
	Turmeric	• Contains curcumin antioxidant to reduce inflammation<br>• May increase short-term memory and prevent formation of brain tangles that cause dementia
	Honey	• Some anti-bacterial and anti-viral properties<br>• Antioxidants<br>• May lower risk of heart disease and diabetes by lowering cholesterol, triglyceride levels and blood sugar levels
	Cinnamon	• Contains polyphenols and cinnamaldehyde which prevent dementia<br>• Polyphenols reduce inflammation and protect against toxins<br>• Cinnamaldehyde prevents formation of brain tangles that cause dementia
	Hemp oil	• Contains essential fatty acids and omega-3's (for brain and heart health) and important amino acids
	Skim milk powder	• Contains protein (muscle health) and calcium and vitamin D (bone health)
